# Shared Psychotic Manic Syndrome in Monozygotic Twins: A Case Report

**Published:** 2012

**Authors:** Mohammad Reza Ghasemzadeh, Mohammad Ghadiri Vasfi, Shabnam Nohesara, Amir Shabani

**Affiliations:** 1 Psychiatric Resident, Mental Health Research Centre, Tehran Psychiatric Institute, Tehran University of Medical Sciences, Tehran, Iran.; 2 Assistant Professor of psychiatry , Mental Health Research Centre, Tehran Psychiatric Institute, Tehran University of Medical Sciences, Tehran, Iran.; 3 Associate Professor of psychiatry, Mental Health Research Centre, Tehran Psychiatric Institute, Tehran University of Medical Sciences, Tehran, Iran

**Keywords:** Identical twin, Mania, Shared Psychotic Disorder

## Abstract

**Objective: **A rare phenomenon of Shared Psychosis Disorder occurring in the context of Bipolar I Disorder, in identical twins is reported.

**Case Presentation:** Two identical twins with shared Psychotic Manic Syndrome were admitted and received antipsychotic and lithium as their treatment. Psychotic symptoms of primary case did not improve and her diagnosis changed into Schizophrenia. They had hypothyroidism at the same time.

**Conclusion: **Completely shared manic syndrome along with the psychotic features shows a need for the criteria of shared syndromes to develop, including both psychotic and mood symptoms.

## Introduction

Shared Psychotic Disorder or Induced Psychotic Disorder was first described by Lasègue and Falret in 1877 (1). It was also known as folie à deux (2). Diagnostic and Statistical Manual of Mental Disorders, 4th edition, Text revised (DSM- IV -TR) defines it as “a delusion develops in an individual in the context of a close relationship with another person or persons, who have an already established delusion and it is similar in content to that of the person who already has an established delusion” (3). It is an unusual syndrome in which a primary case transfers his/her delusional beliefs to secondary case (4) and usually delusions disappear when separation occurs (5). Most patients are members of the same family (6). Shared psychotic disorder among patients suffering from bipolar disorder is very rare (5). 

## Case Report

Two sisters presented here as A and B were brought to the emergency department of a university affiliated psychiatric hospital on December 2009, complaining that they both have stopped eating food. They were 22 year old identical twins and both were single and studying at college. They lived with their parents and their brother. According to family, twin A was the dominant (primary) one in their close relationship. Interview with family revealed that‚ twin A was well until September 2009 when she developed aggression followed by a decrease in need for sleep (2-3 hours in 24 hours), decreased appetite‚ increased religious activities, irritability and walking around. Upon assessment of her mental status she had elevated mood‚ grandiosity delusion stating that she has a lot of money in Swiss banks‚ religious delusion about being in connection with Imams and she should fast the same way they do. Also she had auditory and visual hallucinations such as hearing the voice of Imams and seeing them. Furthermore she had become isolated socially and had no interpersonal relationships.

Twin A^’^s sister (twin B) was well until November 2009 when she developed aggression gradually. Then she became irritable, hyper religious, and her need for sleep was considerably reduced (3 hours in 24 hours). Her appetite decreased and she spent lots of time walking around. In mental status examination, she appeared distractible; she had elevated mood and grandiosity delusions about being connected to Imams, for instance she should fast as they do. She also had auditory and visual hallucinations stating that she hears the voice of Imams and sees them. She did not have any interpersonal relationships and was socially isolated.

Neither our patients nor their family had any history of past psychiatric or medical disorder. They were admitted in two separate wards and their routine laboratory tests and brain imaging were normal. According to DSM-IV-TR and the interviews done by two psychiatrists, they were diagnosed as Bipolar I Disorder, Manic Episode with psychotic features.

Because of severity of their disorder, they were put on lithium, haloperidol and a benzodiazepine. They had good drug compliance. After three weeks of hospitalization mood -related symptoms of both twin A and twin B improved. In addition, Psychotic symptoms of twin B improved while psychotic symptoms of twin A persisted.

One month later, they both were discharged and Because the diagnosis of Bipolar I Disorder and similarities to Shared Psychotic Disorder, they received previous drugs as maintenance treatment and the separation was also recommended; twin A went back home and twin B went to her aunt^’^s house. During follow up period, psychotic symptoms of twin A continued despite being treated with antipsychotics. In September 2010, both twins A and B developed hypothyroidism. Therefore their Lithium was discontinued and they were put on sodium valproate and levothyroxine.

 Because of persistence of psychotic symptoms in A during one year of follow up and only three weeks of mood symptoms the diagnosis changed into schizophrenia.

## Discussion

Similar rare cases have been reported until now (6), but the interesting point in this case report is the completely shared manic syndrome along with the psychotic features. The literature review shows that only two cases with these characteristics have been reported. In one report, two identical twin sisters with positive history of bipolar disorder showed shared delusions, following relapse of the manic symptoms (7) and in the other report, two sisters, showed shared manic syndrome with psychotic features at the same time (5).

 Our case report is similar to the other cases of shared psychotic disorder, because this syndrome often occurs among the members of the same family (6) and most commonly between sisters (8).

Since delusions were transferred from twin A to twin B, and also because of the persistence of psychotic symptoms in twin A in spite of separation, she was considered the primary case. In the other studies it was noted that 72٪ of primary cases and 54٪ of secondary cases were females (8). In Shared Psychotic Disorder the most common transferred symptoms, are delusions and the most common delusions are persecutory (9) which were absent in our patients. But religious and grandiosity delusions that were shared in our cases were reported in 27٪ and 2-13٪ of the cases respectively (10).

In our patients, several symptoms of a manic episode, such as elevated mood, decreased need for sleep, distractibility, hyper religiosity, hyperactivity and grandiosity delusions were transferred, while single transferred symptom of excitement, only notes in 2% of the cases with shared psychotic disorder (7).

Multiple factors such as environmental (closed emotional relationships) and genetic vulnerability factors could be important to create Shared Psychotic Disorder (11). Manifestation of the symptoms in these sisters, with a one month interval, can be explained not only because of the closed emotional relationships between the two sisters but also due to their genetic overlap. On the other hand, vulnerable genetic overlap is seen in schizophrenia and bipolar disorder (12, 13). 

Hypothyroidism was not detected before medical treatment and no positive family histories for hypothyroidism were reported. However 9 months after the onset of lithium in our patients, both of them showed hypothyroidism at the same time. Findings from genetic-association studies have shown that there are lithium responsive genes in bipolar disorders (14) and it might be assumed that there is a genetic susceptibility in lithium- induced hypothyroidism.

In DSM-IV-TR and International Statistical Classification of Diseases and Related Health Problems, 10th Revision (ICD-10) criteria, the transfer of shared psychotic symptoms are discussed but not induced mood disorder or simultaneous transfer of mood symptoms (3,15), therefore such cases will have no specific diagnoses. 

At the end, it should be noted that there are no criteria in DSM-IV-TR and ICD-10 for shared mood symptoms, so there is a need for developing criteria of shared syndromes , including both psychotic and mood symptoms. 

## Authors’ Contributions:

MRGh drafted the manuscript. MGhVasfi and ShN have been involved in the acquisition of clinical data. ASh read and edited the manuscript. All authors read and approved the final manuscript.

## Notes

**Declaration of interest: **None
